# Center-of-gravity shift and inequality of human water use in China over the last half century

**DOI:** 10.1038/s41598-026-42569-x

**Published:** 2026-03-03

**Authors:** Yanbo Zhao, Qimin Ma, Jixiang Jia

**Affiliations:** 1 National Cryosphere Desert Scientific Data Center, Lanzhou, 730099 China; 2 Northwest Institute of Eco-Environment and Resources, Chinese Academy of Sciences, Gansu, 730000 China; 3 College of Resources and Environment, Chengdu University of Information Technology, Chengdu, 610225 China

**Keywords:** Human water use, Spatial dynamics, Water use inequality, Gravity movement, Gini coefficient, China, Environmental sciences, Environmental social sciences, Hydrology, Water resources

## Abstract

Understanding the spatial dynamics and inequality of human water use is essential for achieving sustainable water management. Although previous studies have examined the spatial evolution of regional water use, comprehensive assessments that couple sectoral decomposition with long-term (> 50 years) national-scale dynamics remain limited. To address this gap, we compiled multi-sectoral water use data from 1970 to 2020 and analyzed spatial trends and inequalities in China’s water use using the center-of-gravity approach and Gini coefficients. Our results reveal distinct directional shifts among sectors: irrigation and industrial water use moved northeastward and southwestward, respectively, while total water use exhibited minimal spatial displacement. Water-use inequality declined from high to moderate levels before 2000 and has since stabilized. Irrigation water use dominated both the spatial redistribution and inequality of total water use, driven primarily by changes in irrigated area and industrial scale. Inter-provincial disparities accounted for the largest share of overall water-use inequality. These findings offer new insights into the spatial evolution of China’s water use and provide an empirical basis for promoting more equitable and sustainable water allocation policies.

**Supplementary Information:**

The online version contains supplementary material available at 10.1038/s41598-026-42569-x.

## Introduction

Water is a vital resource essential for societal and economic activity^1^. The past century has seen rapid population growth, increased economic activity, and higher living standards, increasing global water use by nearly eight times from 500 km^3^ yr^− 1^ to 4000 km^3^ yr^[− 12^. This increase in demand has led to water stress in many nations^3^, with about one-third of the global population facing water scarcity^4^. Meanwhile, severe environmental problems, such as groundwater depletion, decreased wetlands and lakes, and weakened river connectivity, has been induced in many regions of the world^5–8^, such as North China Plain, the Indus River basin, and the Great Plains of the United States. In such a context, understanding the spatiotemporal trajectories of human water use and their driving factors becomes extremely important for sustainable water resources management.

Water problems are particularly challenging in China owing to its large population, fast-growing economy, inadequate governance, and uneven water distribution^9,10^. Many studies have analyzed the temporal changes and socioeconomic drivers of China’s water use^11–17^. These investigations demonstrated an upward but decelerating trend in water use until 2012, followed by a decline in water use due to the implementation of the strictest water resources management system (SWRMS)^16,18^. Furthermore, socioeconomic development and improvements in water use efficiency were found to have conflicting impacts on water use changes. Nevertheless, there is a notable gap in the exploration of spatial dynamics in water usage. Such knowledge is critical for macro water management and decision making^19^. As a result, the trends and drivers of spatial changes in water use in China remain unclear.

The center of gravity is a quantitative measure of spatial balance in mass distribution, with its movement reflecting shifts or accumulations in spatial patterns^20^. Accordingly, the concept of a “center of gravity” provides an effective framework for characterizing the spatial dynamics and redistribution of socioeconomic activities and environmental changes^21–23^. This approach has been widely applied to explore the spatial evolution of diverse phenomena, including GDP, carbon emissions, and land use^16,24–28^. Several studies have further adopted the center-of-gravity method to examine the spatial evolution of regional total water use^13,29^ and sectoral water-use dynamics^30^. However, these efforts have largely overlooked the decomposition of water-use shifts by sector—such as industrial, domestic, and irrigation water use—and have not identified the underlying drivers through long-term, nationwide assessments. Our study adds new value beyond existing literature by attributing shifts in the water-use gravity center to changes in sectoral water-use scales (irrigated area, industrial output, and population) and intensities (e.g., water use per irrigated area, per unit industrial output, and per capita water use).

Besides the spatial dynamics of water use, ensuring equality in water use, wherein every individual has access to sufficient water for personal and economic activities such as farming, manufacturing, and services, is crucial for promoting sustainable socioeconomic development^31,32^. China experiences a significant disparity between its water resources and population, with uneven distribution patterns leading to pronounced inequality in water use^33,34^. For instance, while North China accounts for only 20% of China’s water resources, it supports over half of its population^35^. Given that large-scale physical interventions are approaching their limits, a comprehensive understanding of water use inequality is crucial for developing multifaceted policies that extend beyond physical regulation to include virtual water trade and demand-side management^36,37^. Several studies have investigated water use inequality in China, revealing important policy implications^36–40^. However, most of these studies have focused on a relatively short time frame in the twenty-first century and have been restricted to a province-level analysis that may underestimate water use inequality. As a result, little is known about the long-term dynamic evolution and sources of water use inequality in China.

To address these gaps, this study utilizes long-term (1966–2020), high-resolution (prefecture-level), and multi-sectoral water-use data to investigate the spatial dynamics and inequality of human water use across China. Specifically, we aim to answer four key questions: (a) What are the spatiotemporal trajectories of water use in China over the past five decades? (b) How have different drivers and regions contributed to these trajectories? (c) How has water-use inequality evolved over time? and (d) What are the dominant sources of this inequality, particularly the relative roles of interregional disparities and prefecture-level water-use intensity? By elucidating these spatial patterns and underlying drivers, our study provides empirical evidence and policy-relevant insights for improving water resource management and advancing sustainable development.

## Materials and methods

**Fig. 1:**
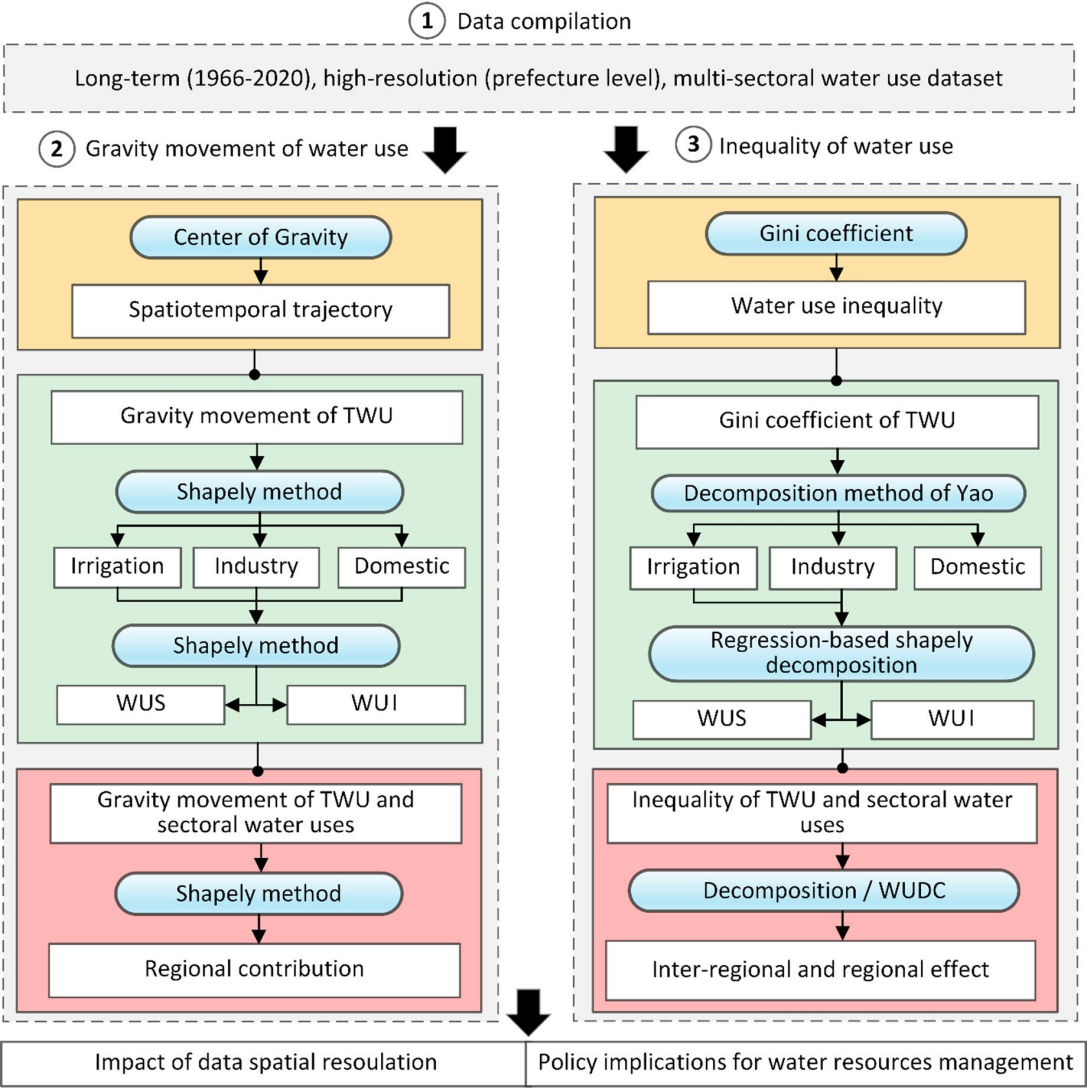
Workflow of this study. The numbered and light gray boxes signify the major steps involved in the study, while white boxes represent datasets, inputs, or outputs. Analysis tools are denoted by blue boxes. The three sub-steps of the second and last major phases are highlighted by green, yellow, and red boxes, respectively. WUS and WUI refer to water use scale and water use intensity, respectively.

Figure 1 illustrates the overall methodological framework of this study. Initially, we compiled a comprehensive prefecture-level, multi-sectoral water-use dataset for China spanning the period 1966–2020. We then employed the center-of-gravity approach to characterize the spatiotemporal trajectories of both total and sectoral water use. To quantify the driving forces behind these trajectories—specifically the relative contributions of water-use scale, intensity, and regional influences—we applied the Shapley decomposition method. Subsequently, the magnitude and temporal evolution of water-use inequality were evaluated using the Gini coefficient, with sectoral contributions estimated via the decomposition framework proposed by Yao^41^. Furthermore, a regression-based Shapely decomposition approach^42,43^ was utilized to disentangle the impacts of per capita water-use scale and water-use intensity (WUI) on the inequality of irrigation and industrial water use. Domestic water use was excluded from this specific decomposition, as its inequality is entirely attributable to intensity when population serves as the scale variable. Finally, we evaluated inter- and intra-regional effects, as well as prefecture-level influences on overall inequality, using decomposition analysis and the Water Use Demand Coefficient (WUDC).

### Datasets

The datasets used in this study fall into two categories: water-related data and socioeconomic data. The water-related data include human water use and irrigated area. We compiled prefecture-level human water use data for 340 prefectures across China covering the period 1966–2020. The datasets for 2000–2020 were collected from the Water Resources Departments of the 31 provinces, local statistical bureaus, and the National Tibetan Plateau Data Center (http://data.tpdc.ac.cn/en/) (Supplementary Table S1). These datasets are derived from a rigorous hybrid statistical system that integrates three distinct data collection methods: continuous monitoring at key hydrological control points for large-scale withdrawals; direct reporting and metering for major irrigation districts, industrial enterprises, and urban utilities; and quota-based estimation for distributed agricultural and rural domestic sectors. This methodology ensures national consistency and reliability, representing the most authoritative water use records in China^44,45^.The dataset for 1966–1999 was obtained from Zhou et al.^16^, who reconstructed a nationwide, survey-based dataset to assess the socioeconomic drivers of China’s water use changes.

Both datasets consistently include water use in the irrigation, industrial, and domestic (urban and rural) sectors, which together account for over 91% of total blue water withdrawal (Supplementary Table S2). In this study, water use refers to the total volume of water withdrawn from rivers and aquifers for human activities, defined as the sum of irrigation, industrial, and domestic uses. It is noteworthy that ecological water use has expanded rapidly in recent years due to national policies such as ecological water replenishment and river restoration (Supplementary Fig S1). However, its contribution to total water use remains limited (approximately 2% during 2010–2020) (Supplementary Fig S2). Moreover, consistent and long-term prefecture-level records of ecological water use are not yet available, which restricts its inclusion in the present analysis.

We obtained prefecture-level irrigated area from multiple sources^46,47^, primarily the Provincial Statistical Yearbook, the Rural Statistical Yearbook, the China Statistical Yearbook for Regional Economy^48^, and the China Water Statistical Yearbook^49^. In addition, we obtained prefecture-level socioeconomic data, including gross value added of industrial products (industrial GVA) and population, from provincial statistical yearbooks and the National Bureau of Statistics (https://data.stats.gov.cn/). To account for inflation, we deflated the original industrial GVA using the Producer Price Index for industrial products to express them at constant 2010 price. The water use intensity of the three water use sectors were estimated by dividing irrigation, industrial, and domestic water use by irrigated area, industrial GVA, and population, respectively.

### Tracking spatial dynamics of water use via the center-of-gravity movement

We adopted the concept of “center of gravity” to track the spatiotemporal trajectory of TWU and sectoral water uses (i.e., irrigation, industrial and domestic) in China. The gravity center of water uses (*X*, *Y*) is represented as:

1 $${X}^{t}=\frac{{\sum}_{i=1}^{n}{WU}_{i}^{t}\times{x}_{i}}{{WU}_{i}^{t}}$$

2 $${Y}^{t}=\frac{{\sum}_{i=1}^{n}{WU}_{i}^{t}\times{y}_{i}}{{WU}_{i}^{t}}$$

where ${WU}_{i}^{t}$ denotes the water use in prefecture *i*; and *x*_*i*_ and *y*_*i*_ are the longitude and latitude of geometric center in prefecture *i*, respectively; and *t* is year. Following the study of Zeng and Ren^50^, we estimate the center of gravity by taking the average water use over a period of five years. For instance, the year 1970 serves as a representative year for the period between 1966 and 1970. This rule applies to all of the following content. The gravity centers serve as an indicator of the concentration of water use, and the time series of gravity centers, calculated from continuous records, illustrate the spatial dynamics of water use over time. The center-of-gravity movement of water use from t1 to t2 can be expressed as:

3 $$\overrightarrow{g}=\left(\varDelta X,\varDelta Y\right)=\left({X}^{\mathrm{t}2}-{X}^{\mathrm{t}1},{Y}^{\mathrm{t}2}-{Y}^{\mathrm{t}1}\right)$$

where the positive direction of longitude is defined as eastward and the positive direction of latitude is defined as northward. For instance, $\overrightarrow{g}=\left(1,1\right)$ denotes that the center of gravity moves towards the northeast, while $\overrightarrow{g}=\left(-1,-1\right)$ indicates that the center of gravity moves towards the southwest. The moving distance of gravity centers from t1 to t2 is calculated as:

4 $$D=R\times\sqrt{{({X}^{\mathrm{t}2}-{X}^{\mathrm{t}1})}^{2}+{({Y}^{\mathrm{t}2}-{Y}^{\mathrm{t}1})}^{2}}$$

where *R* indicates the coefficient of conversion of geographic coordinates into plane distance^25^, which is approximately equal to 111 km.

### Driving factors and regional contributions to gravity movement

We applied the Shapely method to quantify the contribution of different driving factors and regions to the gravity movement of water use. The Shapley method is a cooperative game theory solution concept that was originally proposed by Shapley^51^ to allocate the total reward earned by a group of players among themselves by measuring the marginal contribution of each player to the total reward. The average marginal contribution of the player *i* (i.e., the Shapely value of the player *i*) can be estimated using Eq. 5, with the consideration of all possible orderings of the players.

5 $${S}_{i}\left(\mathrm{v}\right)=\sum_{O\in\pi\left(n\right)}\frac{1}{n!}(v\left({Pre}^{i}\left(O\right)\cup i\right)-v({Pre}^{i}\left(O\right)\left)\right)$$

where *O* is one of the possible orderings of the *n* players; $\pi\left(n\right)$are all the possible orderings of the *n* players; *v* is the value function of the jointed players; and ${Pre}^{i}\left(O\right)$ is the predecessors of the player *i* in the order *O*.

In this study, the value function employed is the gravity movement along the direction of longitude (i.e., ΔX) or latitude (i.e., ΔY). We first quantify the contribution of sectoral water uses (i.e., irrigation, industrial and domestic) to the gravity movement of TWU. As there are three players (sectors), there are six possible orderings. The marginal contributions of each sector to the gravity movement of TWU along the directions of longitude and latitude are calculated using Eqs. 6 and 7, respectively. The marginal contributions of each sector are further projected to the direction of TWU movement using Eq. 8.

6 $${S}_{i}\left({\Delta}\mathrm{X}\right)=\sum_{O\in\pi\left(3\right)}\frac{1}{3!}\left({\Delta}\mathrm{X}\left({Pre}^{i}\left(O\right)\cup i\right)-{\Delta}\mathrm{X}\left({Pre}^{i}\left(O\right)\right)\right)$$

7 $${S}_{i}\left({\Delta}\mathrm{Y}\right)=\sum_{O\in\pi\left(3\right)}\frac{1}{3!}({\Delta}\mathrm{Y}\left({Pre}^{i}\left(O\right)\cup i\right)-{\Delta}\mathrm{Y}({Pre}^{i}\left(O\right)\left)\right)$$

8 $${MC}_{i}^{g}=\frac{\overrightarrow{{a}_{i}}\times\overrightarrow{g}}{\left|\overrightarrow{g}\right|}$$

where $\overrightarrow{g}$ is the gravity movement of TWU, and $\overrightarrow{{a}_{i}}=({S}_{i}\left({\Delta}\mathrm{X}\right),{S}_{i}({\Delta}\mathrm{Y}\left)\right)$.

The Shapley method was also used to decompose the contribution of driving factors to the gravity movement of sectoral water uses. For each sector, the water use was decomposed into water use scale and water use intensity, respectively. Specifically, irrigation water use was decomposed into irrigated area and irrigation WUI; industrial water use was decomposed into industrial GVA and WUI; and domestic water use was decomposed into population and domestic WUI. In this case, the estimates of Shapley value only involve two players, resulting in two possible orderings. Lastly, the contribution of each province to the gravity movement of water use was estimated. As there are 31 players involved, there are 31! = 8.2 × 10^35^ possible orderings, leading to a considerable computational burden. To resolve this problem, the polynomial method based on sampling theory was used to estimate the Shapley value. The key idea behind this method is to use the sample mean as an estimate of the population mean^52^. In this study, 10,000 samples were taken from all possible orderings of the provinces to estimate the average marginal contribution of each province to the gravity movement of water use.

### Measuring inequality of water use via the Gini coefficient

The Gini coefficient, commonly utilized to evaluate income or wealth inequality, was employed in this study to assess the degree of inequality in water use across prefectures in China. The Gini coefficient offers an advantage over other inequality measures (e.g., standard deviation) as it can be decomposed into different components or sources, allowing for a quantitative assessment of the factors driving inequality^36^. The Gini coefficient is defined based on the Lorenz curve (Supplementary Fig S3), which plots the cumulative share of water use against the cumulative share of the population, ordered by per capita water usage. The Lorenz curve is then compared to the line of perfect equality, and the Gini coefficient is calculated as the ratio of the area between the two curves to the total area below the line of perfect equality. The Gini coefficient ranges from 0 to 1, with higher values indicating greater inequality. We estimated the Gini coefficients of TWU and sectoral water use using the formula proposed by Yao^41^, as in Eq. 9.

9 $$G\left(t\right)=1-{\sum}_{i=1}^{n}{p}_{i}(2{Q}_{i}-{w}_{i})$$

where $G\left(t\right)$ represents the Gini coefficient for the year *t*; *n* is the number of prefectures in China; *p*_*i*_ is the proportion of population in prefecture *i* to the total population; *w*_*i*_ is the proportion of water use in prefecture *i* to the total water use in all prefectures; and ${Q}_{i}$ represents the proportion of cumulative water use from the first to the *i*^th^ prefecture. To calculate the Gini coefficient, prefectures must be ranked in ascending order according to per capita water use. Based on the Gini coefficient, we classified inequality of water use into four different categories^53^: low (G < 0.30), moderate (0.30 < G < 0.40), high (0.40 < G < 0.50), and severe (G > 0.50). In this study, the term ‘inequality’ refers to the spatial unevenness of water use distribution relative to population. It serves as a statistical metric derived from the Gini coefficient and does not imply a normative judgment of social injustice or inequity. A high Gini coefficient in resource use simply reflect the concentration of water-intensive sectors (e.g., agriculture or industry) in specific regions, which could indicate either potential water stress or high resource use efficiency, depending on the context. Therefore, ‘inequality’ in this manuscript is used as a neutral descriptor of distributional heterogeneity.

TWU represents the aggregate water use across three sectors: irrigation, industry, and domestic. The inequality of TWU was decomposed by sector (see Supplementary Text). Specifically, inequalities in irrigation and industrial water use were further decomposed into a scale effect (i.e., irrigated area or industrial GVA per capita) and an intensity effect (i.e., water use per unit of irrigated area or industrial GVA). Furthermore, we conducted a regional decomposition of the Gini coefficient. To quantify the contribution of prefecture-level water use to overall inequality, we calculated the water use demand coefficient (WUDC), following previous studies^39,53^.

10 $${WUDC}_{i}=\left(\frac{{TWU}_{i}}{\sum_{i=1}^{n}{TWU}_{i}}\right)/\left(\frac{{Pop}_{i}}{\sum_{i=1}^{n}{Pop}_{i}}\right)$$

where *TWU* is total water use; *Pop* is population; *i* is prefecture; and *n* is the number of prefectures in China. A deviation of WUDC from 1 indicates the contribution to overall water inequality, with a larger deviation indicating a greater contribution.

## Results

### Spatial change of water use and its driving forces

#### Center-of-gravity movement of water use

**Fig. 2:**
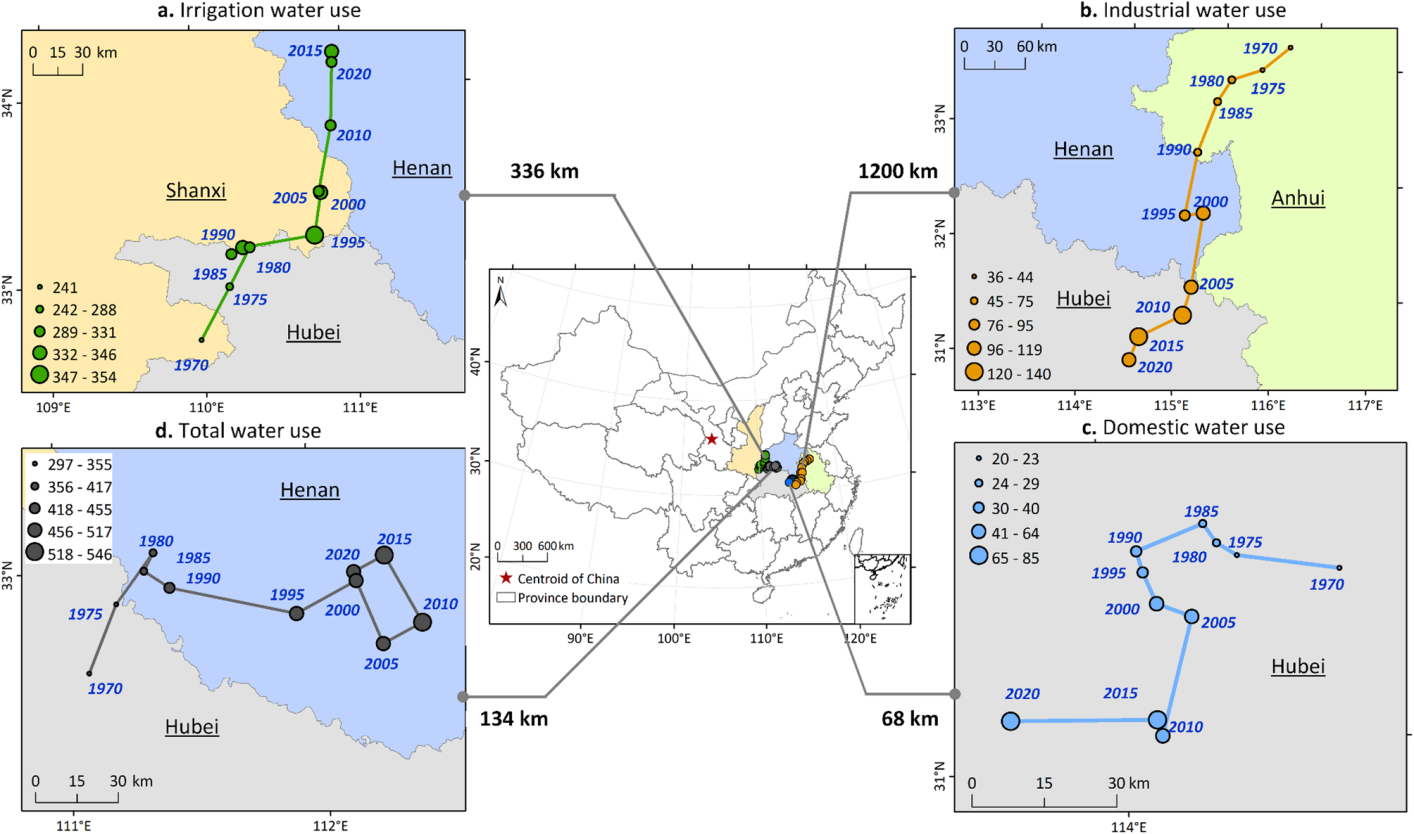
Spatiotemporal trajectories of the center of gravity for total water use (TWU) and sectoral components (irrigation, industrial, and domestic) in China (1970–2020). Numbers next to circles indicate representative years (5-year means). The central map summarizes the net shift distance and location for each sector relative to China’s geometric center. The map was generated using QGIS software, Version 3.40 (https://www.qgis.org).

Figure 2 illustrates the movement of the centers of gravity for total water use (TWU) and its sectoral components—irrigation, industrial, and domestic water use. The center of gravity for irrigation water use exhibited a pronounced northeastward shift, moving approximately 336 km from 1970 to 2020. Although irrigation water use increased substantially between 1970 and 1995, it showed an overall declining trend thereafter. In contrast, the center of gravity for industrial water use shifted markedly southwestward by more than 1,000 km, consistent with the continued rise in industrial water use from 1997 to 2015, followed by a recent decline. These opposite directional shifts in irrigation and industrial water use resulted in only a minor net displacement of the TWU center of gravity—approximately 134 km toward the northeast. Domestic water use exhibited the smallest spatial shift, with its center of gravity moving less than 100 km during 1970–2020. Overall, the centers of gravity for TWU and all sectors remained consistently located southeast of China’s geometric center, reflecting the concentration of socioeconomic activities and intensive water use in southeastern China relative to the northwest (Supplementary Fig S4).

#### Driving factors to gravity movement

**Fig. 3:**
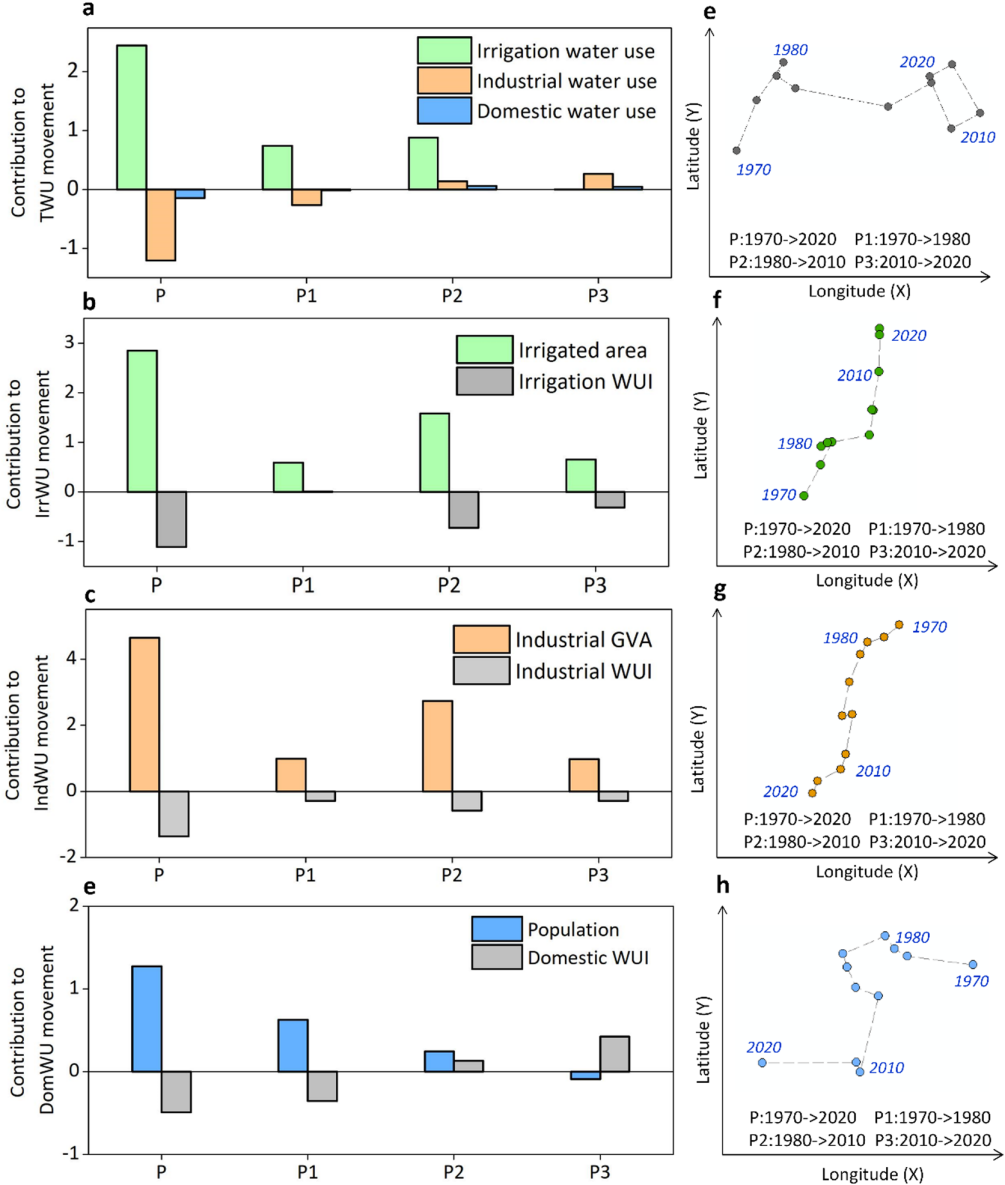
Drivers of gravity center shifts across different periods. **a**–**d** Contributions of driving factors (scale vs. intensity) to the gravity movement of TWU and individual sectors. **e**–**h** Corresponding gravity center trajectories for each sector. Periods are defined as P (1970–2020), P1 (1970–1980), P2 (1980–2010), and P3 (2010–2020). *WUS* Water Use Scale, *WUI* Water Use Intensity.

To quantify the contribution of driving factors to the gravity movement of TWU, we divided the study period (1970–2020) into three sub-periods (P1:1970–1980, P2:1980–2010, P3:2010–2020). Results indicate that irrigation water use had a significant positive effect on the gravity movement of TWU during the periods P and P1 (Fig. 3a), offsetting the negative impact of industrial and domestic water use. However, sectoral water usage consistently made positive contributions during the P2 and P3 sub-periods. Irrigation water use had a greater impact than the other two sectors during the periods P, P1, and P2, attributable to its share of approximately 70% in TWU (Supplementary Fig S5). Nonetheless, during the P3 sub-period, industrial water usage became the primary driver to the gravity movement of TWU.

**Fig. 4:**
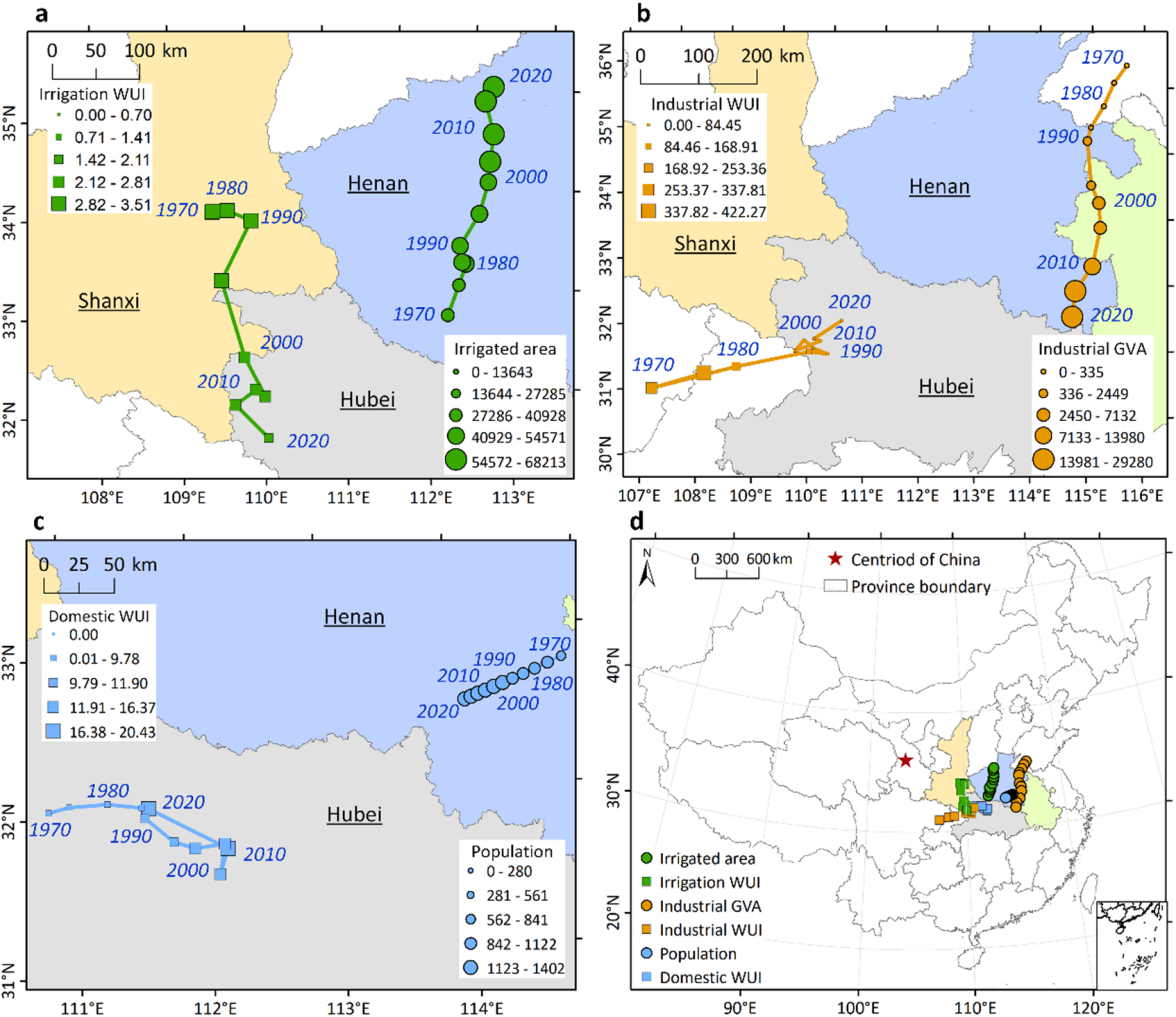
Gravity center trajectories of driving factors (1970–2020). Movements of scale versus intensity factors for **a** irrigation (irrigated area vs. WUI), **b** industry (GVA vs. WUI), and **c** domestic use (population vs. WUI). **d** Overview of gravity center locations for all driving factors. The map was generated using QGIS software, Version 3.40 (https://www.qgis.org).

In Fig. 3b, the contributions of changes in irrigated area and irrigation WUI (i.e., irrigation water usage per unit area) to the gravity movement of irrigation water usage is illustrated. During the P, P2, and P3 periods, irrigated area made a positive contribution while irrigation WUI had a negative contribution. This is due to the northeastward shift of the gravity center of irrigation area, which is similar to that of irrigation water use, while the gravity center of irrigation WUI moved southwestward in the opposite direction, as shown in Fig. 4a. The minimal spatial shift of irrigation WUI led to its limited effect on the gravity movement of irrigation water use during the P1 period.

Industrial GVA shifted southwestward and made a consistent positive contribution to the gravity movement of industrial water use throughout all sub-periods. Conversely, industrial WUI (i.e., water use per unit of industrial GVA) shifted northeastward and had a consistent negative impact. The population shifted slightly southwestward and had a positive contribution to the center of gravity movement of domestic water use during the P, P1, and P2. Domestic WUI (i.e., water usage per capita) did not exhibit a consistent spatial shift and had a positive effect on the gravity movement of domestic water use during P and P1, but a negative effect during P2 and P3.

#### Regional contribution to gravity movement

**Fig. 5:**
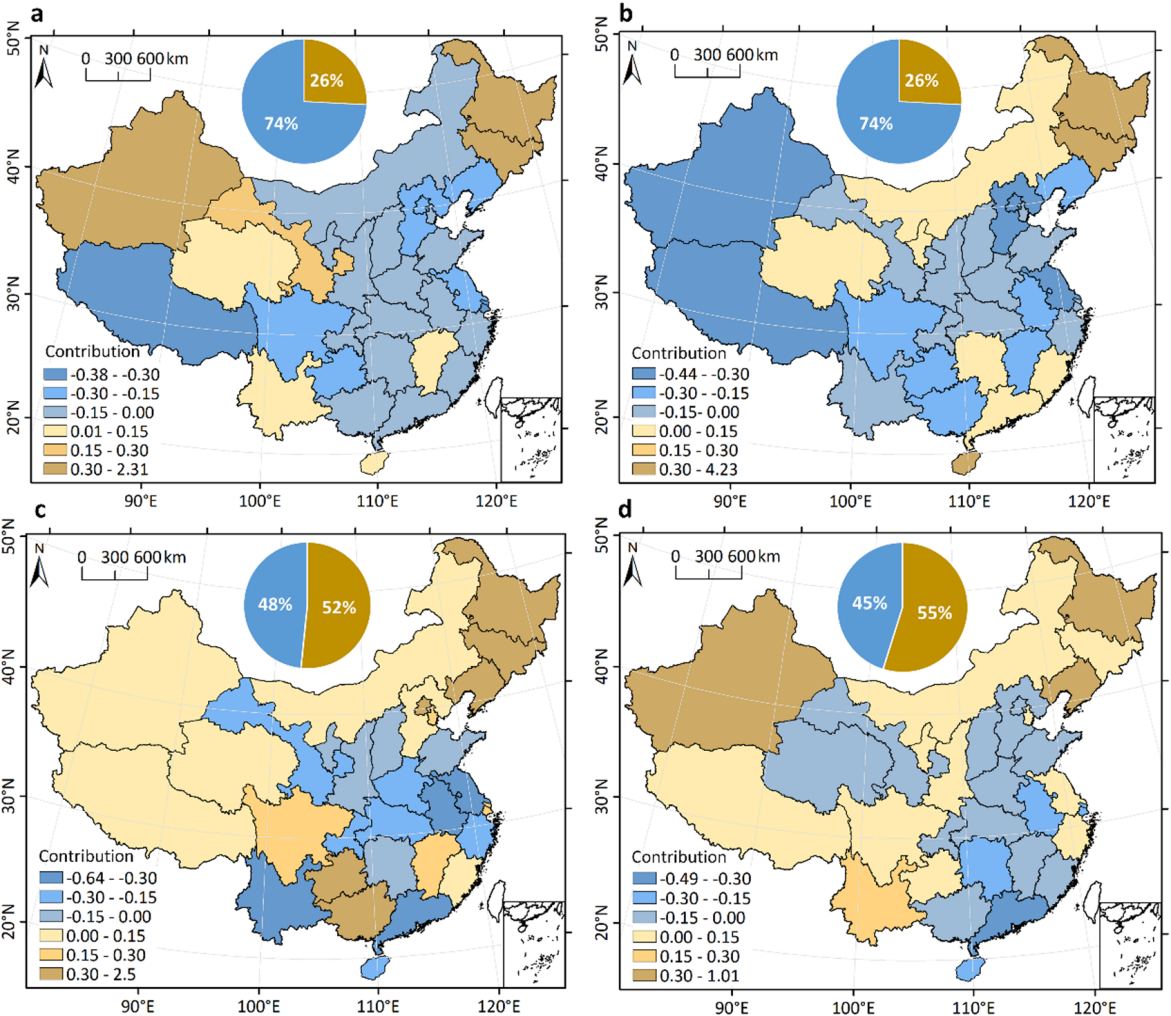
Provincial contributions to the gravity movement of water use (1970–2020). Maps show contributions to the gravity movement of **a** TWU, **b** irrigation, **c** industrial, and **d** domestic water use. Pie charts illustrate the proportion of provinces making positive (yellow) versus negative (blue) contributions. The map was generated using QGIS software, Version 3.40 (https://www.qgis.org).

Figure 5 illustrates the contribution of different provinces to the gravity movement of human water use from 1970 to 2020. A noteworthy observation is that a quarter of the provinces, mainly situated in northwest and northeast China, contributed positively to the northeastern shift of TWU. The remaining 75% provinces had a negative impact, although less pronounced than the positive contributors. Similarly, a quarter of provinces, mainly located in Northeastern and Southern China were the major positive contributing region to the northeastward shift of irrigation water use, which outweighs the effects of the remaining 75% negative contributors. With respect to industrial water use, approximately half of the provinces located in northern China made positive contributions to the southwestward shift in its gravity center, while the other half had negative contributions. The total contribution of the positive contributors was higher than that of the negative contributors. Nearly half of the provinces, primarily located in the northwest, northeast, and southwest of China, had a positive impact on the southwestern shift of domestic water use, while the remaining half has a negative effect.

### Water use inequality and its decomposition

#### Inequality of water use

**Fig. 6:**
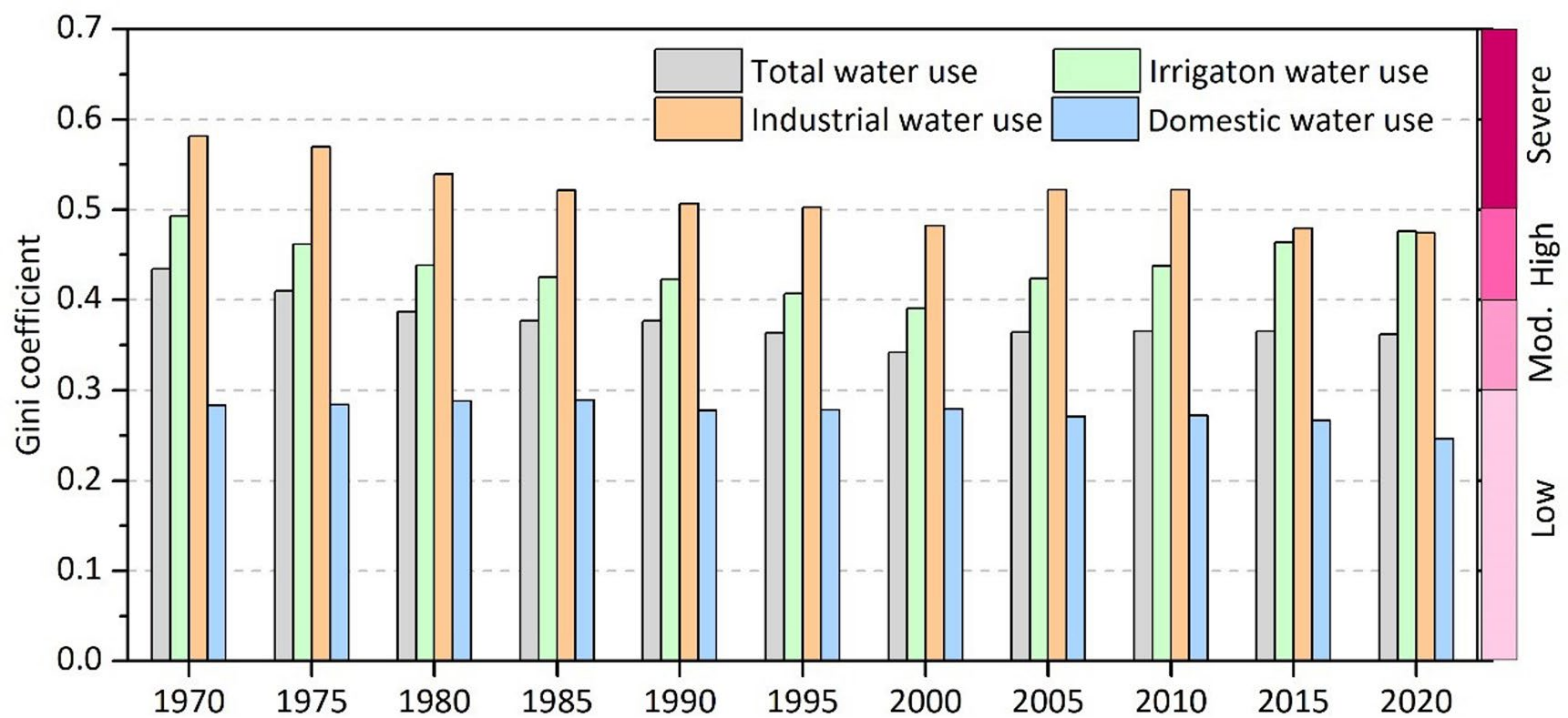
Temporal variations in Gini coefficients (GC) for TWU and sectoral water uses (irrigation, industrial, and domestic) in China from 1970 to 2020. The degree of inequality is represented on the right y-axis, ranging from low inequality (GC < 0.3) to moderate (0.3 ≤ GC < 0.4), high (0.4 ≤ GC < 0.5), and severe inequality (GC ≥ 0.5).

Figure 6 depicts the temporal dynamics of the Gini coefficient for TWU and sectoral water use. Industrial water use exhibits the highest level of water use inequality, with the Gini coefficient indicating a high to severe degree of inequality. Nevertheless, a distinct decreasing trend in the Gini coefficient was observed from 1970 to 2000, followed by an increasing trend from 2000 to 2010, and ultimately a downward trend from 2010 to 2020. Overall, the inequality of industrial water use has significantly reduced over the past few decades. Irrigation water use displays a moderate to high level of inequality, with the Gini coefficient ranging from 0.35 to 0.50. Temporally, the degree of inequality in irrigation water use declined significantly from 1970 to 2000 but reversed to an upward trend afterward. Domestic water use exhibits a low level of inequality, with a decreasing trend over time. The minimal inequality in domestic water use can be attributed to the fact that it is a basic need for everyone, and thus, the government and society allocate and manage water resources more strictly to ensure equal access for all. As for TWU, it exhibits moderate to high inequality, with the Gini coefficient ranging from 0.30 to 0.45. The Gini coefficient of TWU showed a clear decreasing trend until 2000, and has remained relatively stable thereafter.

#### Decomposition of water use inequality

**Fig. 7:**
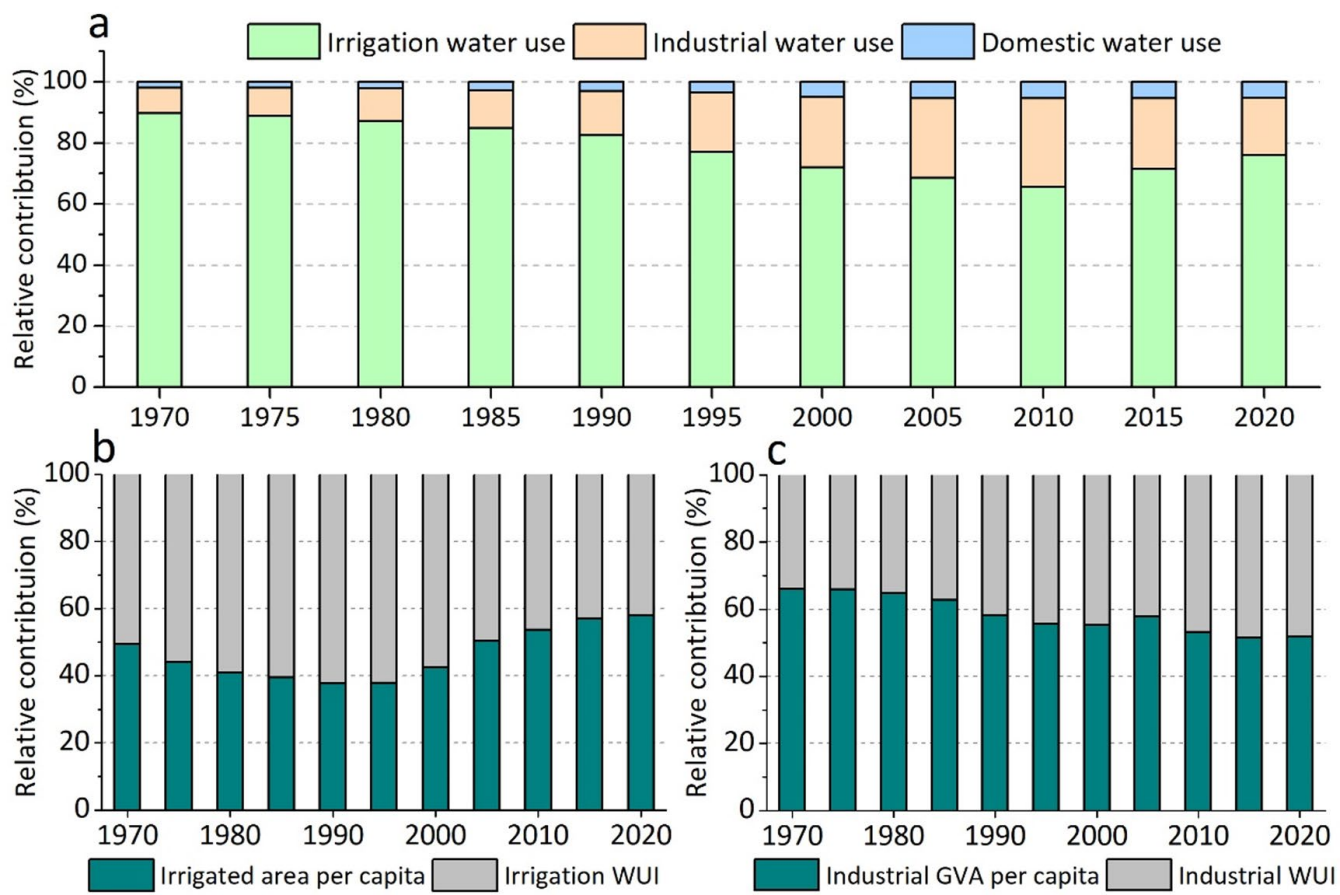
Decomposition of water use inequality (1970–2020). Panel **a** shows sectoral contributions to TWU inequality. Panel **b** illustrates the drivers of irrigation water-use inequality. Panel c illustrates the drivers of industrial water-use inequality.

Figure 7 depicts the decomposition of water use inequality by sector and driving factors from 1970 to 2020. Irrigation water use was the largest contributor to TWU inequality, owing to its substantial share in TWU (Supplementary Fig S5), followed by industrial water use. Notably, the contribution of irrigation water use displays a declining trend from 1970 to 2010, while the contribution of industrial water use shows an upward trend. However, the opposite trend was observed after 2010. These changes in their contribution to TWU inequality can be well explained by their respective shares in TWU (Supplementary Fig S6). Although the contribution of domestic water use to TWU inequality is small, its magnitude is increasing from 1970 to 2020.

The contribution of irrigated area per capita and irrigation WUI to irrigation water use inequality was almost equal in 1970. However, between 1970 and 1995, the contribution of irrigated area per capita decreased to less than 40%, indicating that the uneven spatial distribution of irrigation WUI was the primary driver of irrigation water inequality in 1975. However, after 1995, the contribution of irrigated area per capita started to increase, reaching nearly 60% by 2020, indicating that the mismatch between irrigated area and population has become the main driver of irrigation water inequality today. The contribution of industrial GVA per capita to industrial water inequality was over 60% until 1980 but has been declining since then. At present, industrial GVA per capita and industrial WUI contribute almost equally to industrial water use inequality. These findings suggest that industrial GVA has been distributed more equitably among China’s population from 1970 to 2020.

#### Regional contribution to water use inequality

**Fig. 8:**
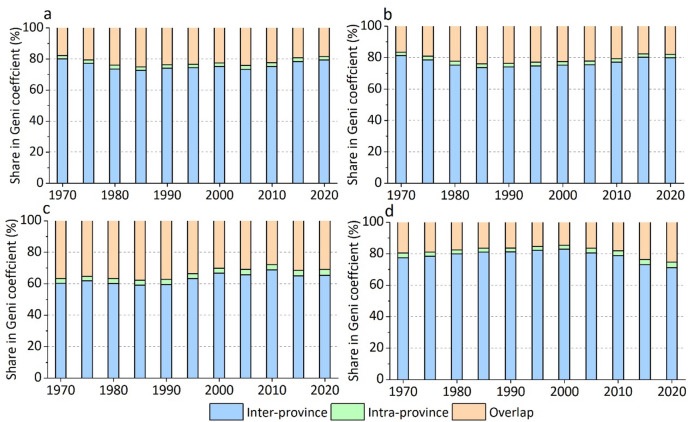
Decomposition of Gini coefficients into inter-province, intra-province, and overlap components (1970–2020). Panels show results for **a** TWU, **b** irrigation, **c** industrial, and **d** domestic water use, respectively.

Figure 8 illustrates the share of inter-province, intra-province, and overlap components in Gini coefficients of water use. The inter-province component is responsible for approximately 80% of the TWU inequality, while the overlapped component accounts for the remaining 20%. In contrast, the intra-province component has a negligible impact on TWU inequality. These findings hold true for industrial water use as well. Regarding irrigation water use, inter-province inequality accounts for approximately 60% of the inequality, with the overlapped effect making up the remaining 40%. Interestingly, the higher overlapped effect for irrigation water use suggests that the average irrigation water use level is more similar among provinces than the average industrial water use level. As for domestic water use, similarly, the inter-province component is the primary driver of inequality, contributing to approximately 80% of the inequality before 2010. However, the overlapped effect has steadily increased from 2010 to 2020, indicating a decreasing degree of stratification of domestic water use among provinces and a higher level of water use equality.

**Fig. 9:**
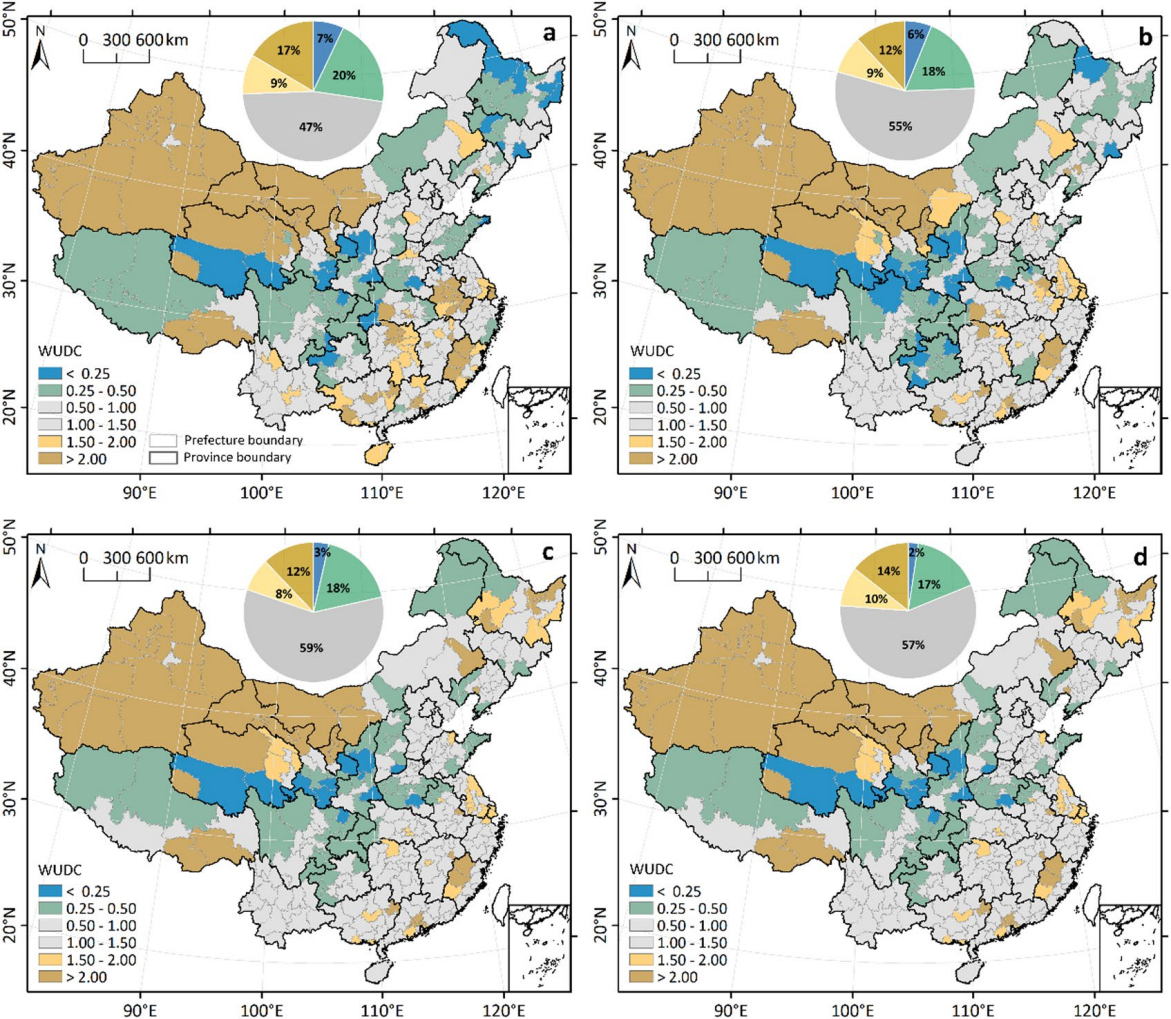
Spatial distribution of the Water Use Demand Coefficient (WUDC). Maps display patterns for representative years **a** 1970, **b** 1985, **c** 2000, and **d** 2020. Pie charts show the percentage of prefectures in each WUDC category. The map was generated using QGIS software, Version 3.40 (https://www.qgis.org).

In Fig. 9, we present the distribution of WUDC for representative years, i.e., 1970, 1985, 2000, and 2020. In 1970, 47% of prefectures had WUDC values between 0.5 and 1.5, indicating that their water use levels were close to the national average and hence contributed less to overall TWU inequality. In contrast, 17% of prefectures (located mainly in northwest and southeast China) had WUDC values above 2, while 7% of prefectures (mainly situated in midwestern and northeastern China) had WUDC value below than 0.25. These prefectures had water uses levels that was significantly greater or less than the national average, thus made a greater contribution to overall water use inequality. The prefectures with WUDC lower than 0.25 accounts for only 0.8% of China’s total water use but accounts for 5.6% of its population. In contrast, the prefectures with WUDC higher than 2.00 accounts for only 7.0% of China’s population but accounts for 27.7% of its total water use. From 1970 to 2020, the number of prefectures with WUDC values between 0.50 and 1.50 increased, with the proportion rising from 47% to 57%. In contrast, the number of prefectures with very low WUDC values (< 0.25) significantly decreased from 7% to 2%. As a result, the overall water use inequality have been decreased. Notably, several prefectures in northeastern China that previously had a lower water use level than the national average shifted to excessive water user after 2000. Meanwhile, the number of prefectures with very high WUDC values (> 2.0) decreased significantly from 1970 to 2020 in southeastern China.

## Discussion

### Impact of data spatial resolution

**Fig. 10:**
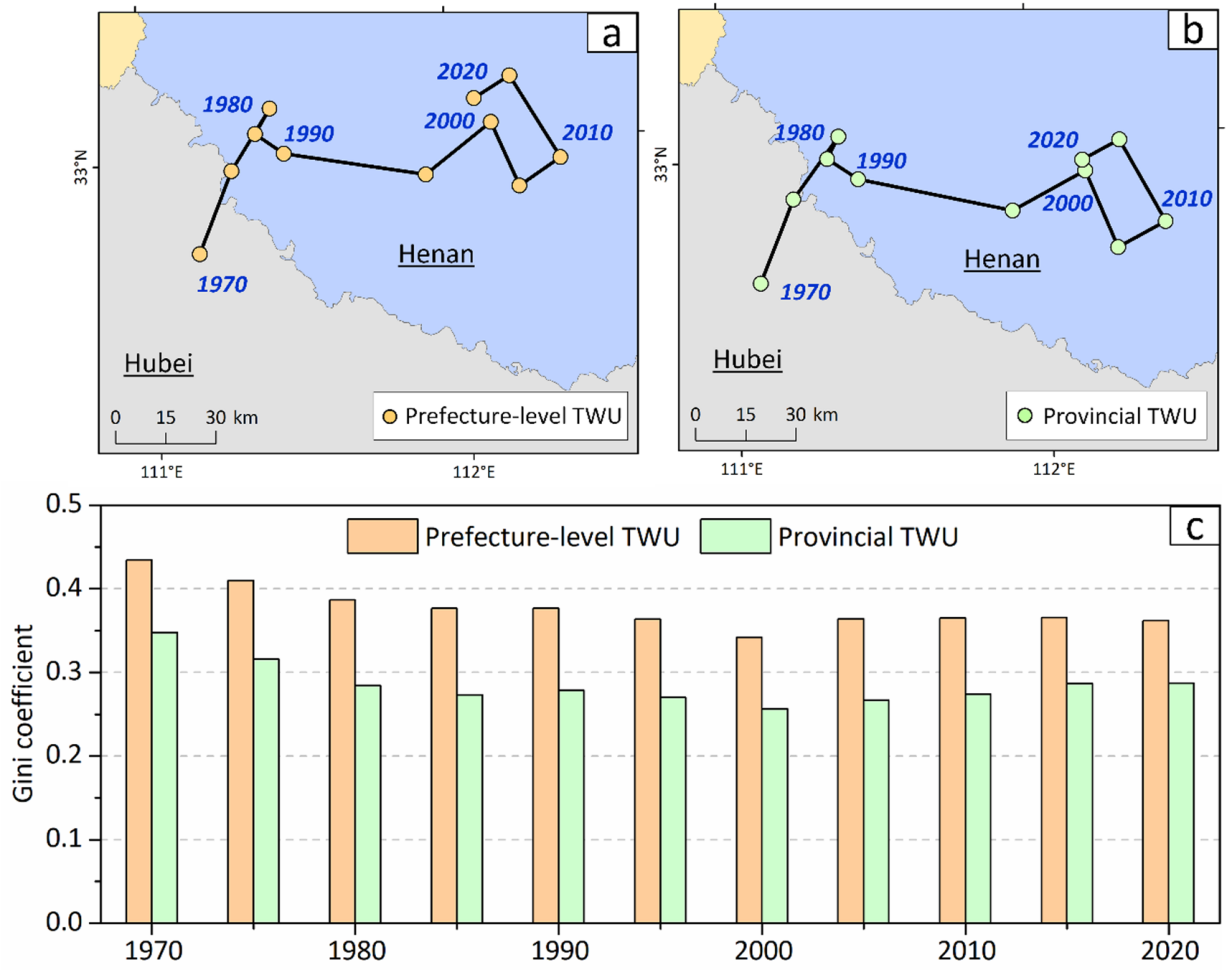
Impact of data spatial resolution on gravity shifts and inequality. Comparison of TWU gravity trajectories using **a** prefecture-level versus, **b** provincial-level data. **c** Comparison of Gini coefficients (1970–2020) derived from the two resolutions. The map was generated using QGIS software, Version 3.40 (https://www.qgis.org).

We further assessed the influence of data spatial resolution by estimating the shifts in the centers of gravity of TWU and the corresponding water-use inequality using both provincial- and prefecture-level data. As shown in Fig. 10, the center-of-gravity movements of TWU are largely consistent across spatial resolutions, indicating that the analysis of gravity-center shifts is relatively insensitive to data aggregation. This consistency arises because provincial TWU represents the sum of prefecture-level values, and a province’s gravity center closely approximates the weighted average of its constituent prefectures. Nevertheless, prefecture-level data remain essential for analyzing water-use gravity movements at finer regional scales.

In contrast, the Gini coefficients for TWU exhibit substantial differences between provincial- and prefecture-level data, with the latter consistently yielding higher values. The relative difference in Gini coefficients ranges from 25% to 38% over the period 1970–2020, suggesting that coarse-resolution data can significantly underestimate water-use inequality, even though both data resolutions capture the overall trend. These findings align with previous studies by Wang et al.^39^ and Sun et al.^36^, who compared city- and provincial-level data for the spatial inequality of China’s water footprint. Wang et al.^39^ reported considerably higher levels of water-use inequality than Sun et al.^36^, highlighting the importance of fine-scale data for accurately assessing inequality.

### Policy implications

This study reveals, for the first time, a pronounced northeastward shift in irrigation water use, primarily driven by spatial changes in irrigated area (Fig. 4). The northeastern and northern regions of China have experienced faster expansion of irrigated areas compared to other regions (Supplementary Fig S7). Croplands in North China have grown substantially over the past decades^54,55^, contributing to a clear northward shift in food production^56^. In Northeast China, fertile black soils, abundant water resources, and a warming climate have facilitated the expansion of rice, soybean, and maize cultivation^57^. Shifts in food consumption patterns have also reinforced this trend: as meat consumption has increased and grain consumption decreased^56^, demand for feed grains—particularly corn, predominantly produced in northern China—has risen^14^. Additionally, investments in water infrastructure, such as canals and reservoirs, have facilitated increases in irrigated area and water productivity in northern regions. Conversely, several southern provinces, including Hunan, Guangxi, Guangzhou, and Zhejiang, have experienced declining irrigated areas. These opposing trends have collectively driven a northward shift in irrigated area and irrigation water use. Given that North China accounts for only ~ 20% of national water resources, this northward movement has exacerbated water scarcity and imposed pressure on fragile ecosystems^58^, with ecological crises reported in regions such as the North China Plain and inland river basins due to excessive irrigation^5,59^. Therefore, targeted management measures—including restrictions on cropland expansion and improvements in water-use efficiency—are essential to ensure sustainable and safe water-food-ecosystem interactions in these regions.

Previous studies suggest that reductions in irrigation water use intensity (WUI), driven primarily by improved efficiency, can offset the water demand growth resulting from irrigated area expansion^14–16^. However, efficiency improvements do not automatically guarantee a reduction in total water withdrawal. As described by the ‘Jevons paradox’, water savings from enhanced efficiency may inadvertently incentivize the expansion of irrigated lands or a shift toward water-intensive crops, thereby offsetting the potential savings^38,60^. Our analysis indicates that irrigated area and irrigation water-use intensity (WUI) exert opposing influences on the gravity movement of irrigation water use. While irrigation WUI has declined across much of North China, it has increased in parts of South China (Supplementary Fig S7), inducing a partial southward shift in irrigation water use. Without the moderating effect of WUI, the center of gravity of irrigation water use would have shifted an additional 175 km northward, further aggravating water stress in North China.

Despite the substantial northeastward shift in irrigation water use, total water use (TWU) has exhibited minimal net spatial displacement over the past decades. This is attributable to the southwestward shift in industrial water use, primarily driven by the spatial redistribution of industrial gross value added (GVA), which has expanded more rapidly in southern China than in northern regions (Supplementary Fig S7). These opposing shifts have effectively stabilized the TWU gravity center: without the influence of industrial water use, TWU would have moved approximately 100 km further north. However, southern China, despite abundant water quantities, continues to experience seasonal water shortages due to inadequate water quality^33^. The southward shift in industrial water use, while mitigating water stress in the north, may exacerbate water quality challenges. Implementation of stringent water-resource management policies, including strict pollutant restrictions in designated water-function areas^18^, will be crucial in addressing these issues.

Population, serving as a proxy for water demand, displayed a subtle southwestward shift over the study period. This pattern may reflect disparities in economic development between western and eastern regions, influencing fertility behaviors, as well as concentrations of ethnic minorities in southwestern China, where family planning policies are relatively relaxed^61^. In contrast, the center of gravity of TWU (a proxy for water supply) shifted northeastward. Consequently, the spatial distance between water demand and supply decreased substantially between 1979 and 2000 (Supplementary Fig S8), indicating a higher degree of alignment and more equitable water use. Since 2000, however, the gravity centers of both population and TWU have remained largely stable, maintaining the same spatial separation.

Our analysis also demonstrates a significant reduction in water-use inequality in China from 1970 to 2000, with relatively stable levels thereafter. Decomposition analysis identifies irrigation water use as the principal contributor to TWU inequality, consistent with previous studies^36,62^, reflecting its dominant share of TWU. Addressing inequality in irrigation water use is therefore critical for reducing overall water-use disparity. Both irrigated area per capita and irrigation WUI substantially influence irrigation water-use inequality and warrant targeted policy attention. The rapid expansion of irrigated area in northwestern and northeastern China has resulted in high water-use demand coefficients (WUDC) in these regions (Fig. 9). Accordingly, a rational reduction in irrigated area in these areas, without compromising food security, would relieve ecosystem pressure and reduce water-use inequality. Furthermore, pronounced spatial heterogeneity in irrigation WUI—even within the same climatic zone (Supplementary Fig S9) —underscores the importance of promoting spatially balanced irrigation practices and water-conservation measures.

While large-scale physical infrastructure projects, such as the South-to-North Water Transfer (SNWT), have played a pivotal role in mitigating regional water scarcity, relying solely on supply-side engineering faces diminishing returns and ecological constraints. As indicated by our results, significant spatial inequality in water use persists. This suggests that future strategies must pivot towards ‘soft’ allocation mechanisms, particularly virtual water trade. By identifying high-inequality hotspots, our study provides the necessary baseline for optimizing regional industrial structures. Regions identified as having high water use intensity but low resource endowment should prioritize importing water-intensive products (virtual water) rather than further exploiting local physical water resources. Thus, the inequality metrics proposed here serve as a crucial diagnostic tool for guiding the transition from physical water transfer to virtual water redistribution.

Finally, it is important to note that this study focuses on human water use—including irrigation, industrial, and domestic sectors—which together account for over 91% of total blue water withdrawals^16^. Although ecological water use has increased in recent years due to policies such as ecological water replenishment and river restoration, its long-term contribution remains limited (~ 2% of total water use during 1966–2020). Given the limited and inconsistent data, ecological water use was not analyzed as a separate sector at the national scale.

## Conclusions

We analyzed the spatial dynamics and inequality of human water use in China (1970–2020) using center-of-gravity analysis, the Gini coefficient, and Shapley decomposition. Results show a northeastward shift in irrigation water use contrasting with a southwestward movement in industrial water use, leading to a minor net displacement in the Total Water Use (TWU) center. Irrigation dominated TWU dynamics until 2010, after which industrial use took precedence. This gravity shift was primarily propelled by the expansion of water-use scale (e.g., irrigated area), while being partially offset by improvements in water-use intensity.

In terms of inequality, the Gini coefficient declined to moderate levels by 2000 and subsequently stabilized. While irrigation remained the primary contributor to inequality, its influence has waned relative to the industrial and domestic sectors. Our decomposition analysis identifies inter-provincial disparities as the dominant source of inequality, highlighting hotspots of disproportionate water use in the northwest and southeast. These results offer a scientific basis for optimizing water allocation strategies and bridging regional gaps in sustainable water management.

## Supplementary Information

Below is the link to the electronic supplementary material.

Supplementary Material 1

## Data Availability

Water use data from 2000 to 2020 is publicly available from the Water Resources Departments of the 31 provinces in China (Supplementary Table S1), while data prior to 2000 can be accessed at https://doi.org/10.6084/m9.figshare.11545176.v1. The code used in this study is available at https://github.com/HydroRS/GravityMove.
