# Supplementary material for: Center-of-gravity shift and inequality of human water use in China over the last half century
Source: Sci Rep. 2026 Mar 3;16:11926. doi: 10.1038/s41598-026-42569-x (PMC13066392; doi:10.1038/s41598-026-42569-x)
Supplement: Supplementary file 1 — Supplementary Material 1 [file 41598_2026_42569_MOESM1_ESM.docx]

# Supplementary Information for

# Center-of-gravity shift and inequality of human water use in China over the last half century

Yanbo Zhao^a,b*^, Qimin Ma^c^, Jixiang Jia^a^,

^a^ National Cryosphere Desert Scientific Data Center, Lanzhou 730099, China

^b^ Northwest Institute of Eco-Environment and Resources, Chinese Academy of Sciences, Gansu 730000, China

^c^ College of Resources and Environment, Chengdu University of Information Technology, Chengdu 610225, China

**Contents of this file**

Texts 1.1

Figures S1 to S9

Tables S1 to S2

References

**Introduction**

This supplementary information (SI) includes one text section, 9 figures, and 2 tables, which provide supplements to the descriptions of the data sets, methods, and results given in the main paper.

# 1. Supplementary Texts

### 1.1 Decomposition of inequality

TWU represents the sum of the water use in the three sectors (irrigation, industry and domestic). The inequality of TWU was decomposed by water use sectors using the method proposed by [Yao^[3]^](file:///Z:\Work_2022\IrrWaterUse\ML_Irr\WU_spatial\Manuscript\Manuscript_Gravity_Movement_V2.docx#_ENREF_42), as in Eq. 1.

$G=\sum_{j=1}^{3} w_{j}C_{j}$ (1)

where *G* is the Gini coefficient of TWU; *w_j_* denotes the proportion of sectoral water use *j* in TWU; *C_j_* is the concentration ration of sectoral water use, which is estimated similarly to Gini coefficient with the difference that prefectures are ranked in ascending order according to per capita TWU, instead of per capital sectoral water use; and$w_{j}C_{j}$ indicates the inequality of TWU that is attributed to sector *j*.

The inequality of irrigation or industrial water use was further decomposed into water use scale per capita (i.e., irrigated area or industrial GVA per capita) and water use intensity (i.e., water use per unit irrigated area or industrial GVA). The regression-based Shapely decomposition approach, which combines a regression model with the Shapley method^[1,2]^, was used to perform the decomposition. The contributions of water use scale per capita (*x*) and water use intensity (*y*) were estimated using Eqs.2 and 3, respectively.

$C\left( x \right)=\frac{1}{2}[(G\left( f\left( x;y \right) \right)-G\left( f\left( \bar{x};y \right) \right)+(G\left( f\left( x;\bar{y} \right) \right)-G\left( f\left( \bar{x};\bar{y} \right) \right)]$ (2)

$C\left( y \right)=\frac{1}{2}[(G\left( f\left( x;y \right) \right)-G\left( f\left( x;\bar{y} \right) \right)+(G\left( f\left( \bar{x};y \right) \right)-G\left( f\left( \bar{x};\bar{y} \right) \right)]$ (3)

where *G* is the function used to estimate the Gini coefficient; *x* is irrigated area or industrial GVA per capita; *y* is water use intensity; and $f\left( x;y \right)=Population*x*y$, and represents the water use function.

Lastly, we conducted a regional decomposition of the Gini coefficient. We first decomposed the Gini coefficient of water use into three components: inter-province ($G_{inter}$), intra-province ($G_{intra})$, and overlap ($G_{overlap})$, as expressed in Eq. 4.

$G=G_{inter}+G_{intra}+G_{overlap}$ (4)

The inter-province component was calculated as the Gini coefficient of water use across all the provinces, while the intra-province component was estimated as the weighted sum of the Gini coefficients of each individual province, as in Eq.5.

$G_{intra}=w_{I}p_{I}G_{I}$ (5)

where *w_I_* is the proportion of water use in province *I* relative to the total water use in all provinces; *P_I_* is the proportion of water use in province *I* relative to the total population; and *G_I_* is the Gini coefficient of water use for province *I.* The overlap component was calculated by subtracting $G_{inter}$and$G_{intra}$from the overall *G* (Eq.6). A higher value of $G_{overlap}$suggests a lower degree of stratification and a greater degree of overlap among provinces, and thus a more equitable distribution of water use.

$G_{overlap}=G-G_{inter}-G_{intra}$ (6)

# 2. Supplementary Figures


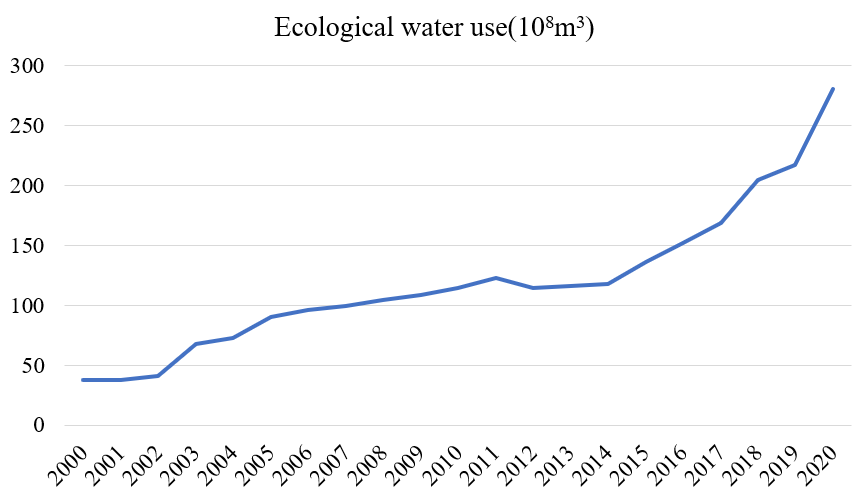


### Fig S1. Changes in ecological water use of China from 2000 to 2020.


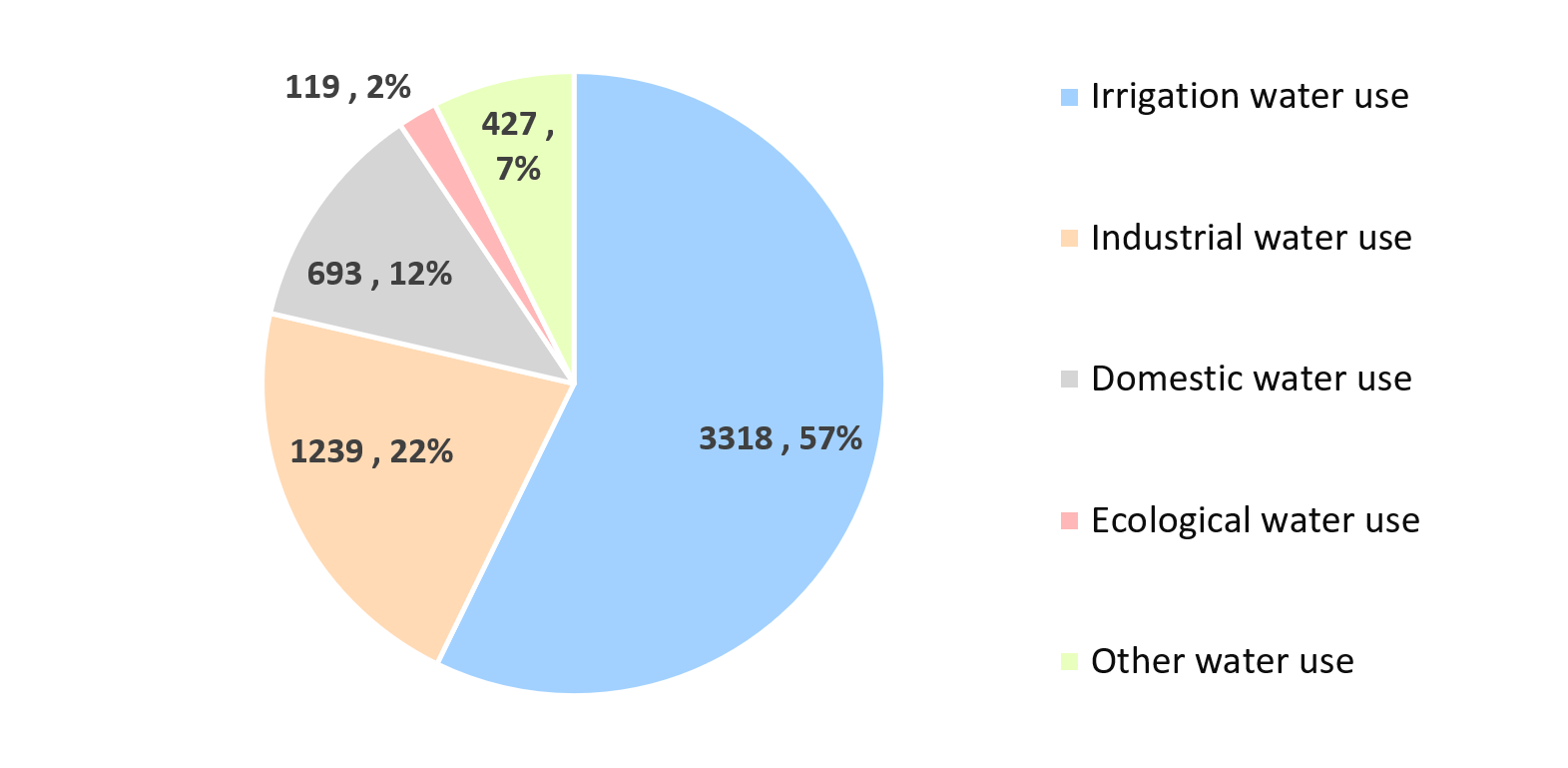


### Fig S2. Proportion (%) of irrigation, industrial, domestic, ecological and other water uses from 2010 to 2020.


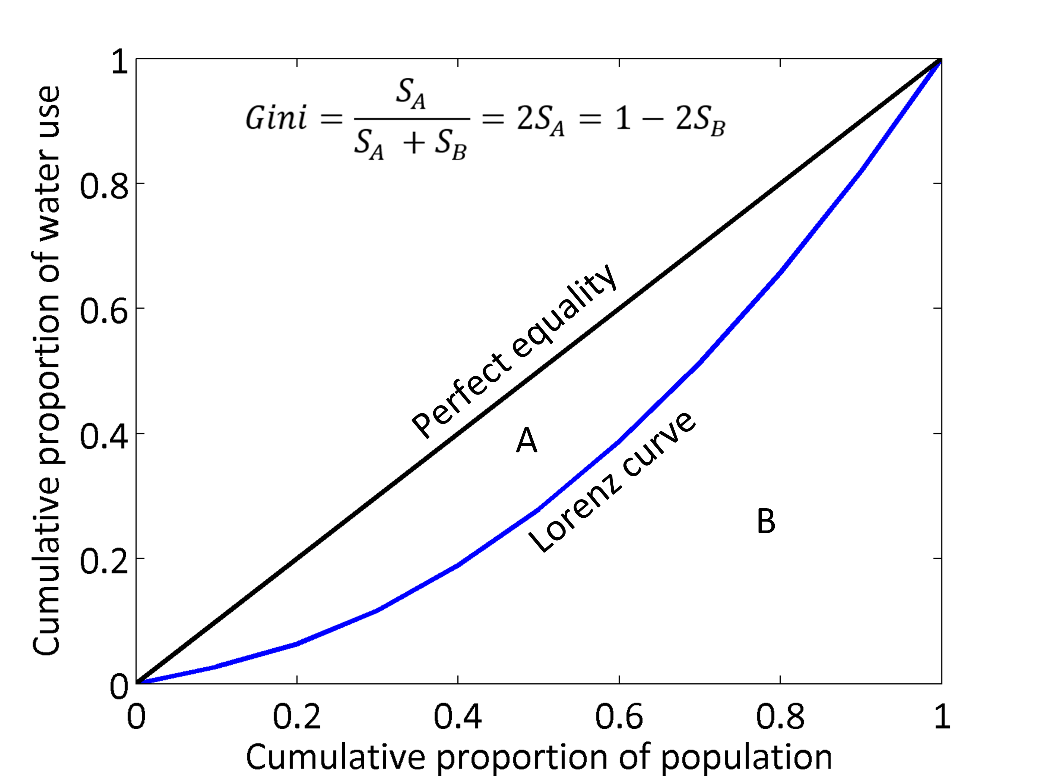


### Fig S3. Schematic diagram of Lorenz curve. The blue line in the plot represents the Lorenz curve, while the black line denotes perfect equality, with a Gini coefficient equal to 0.


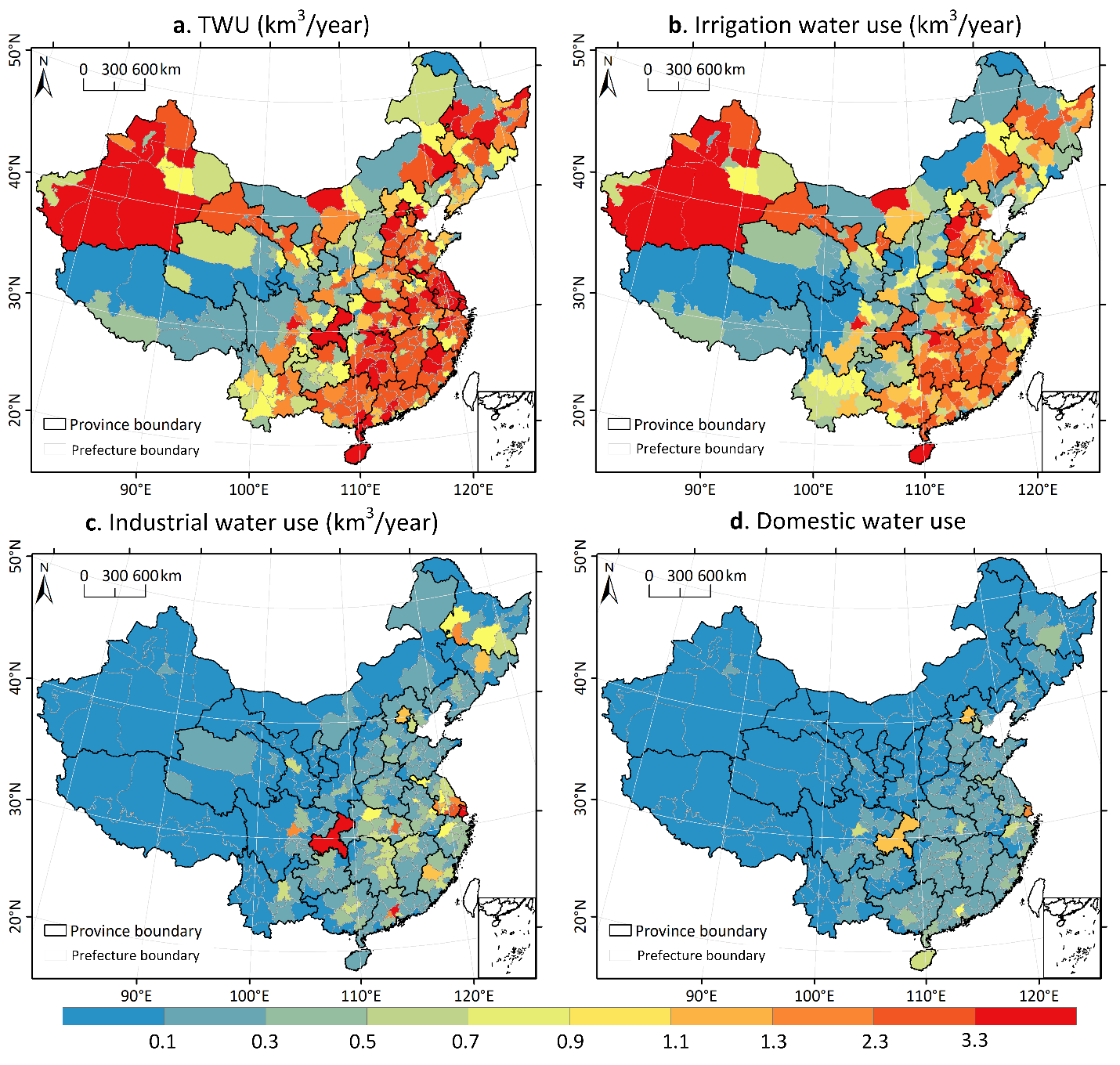


### Fig S4. Spatial patterns of the average TWU and sectoral water use from 1970 to 2020. The map was generated using QGIS software, Version 3.40 (https://www.qgis.org).


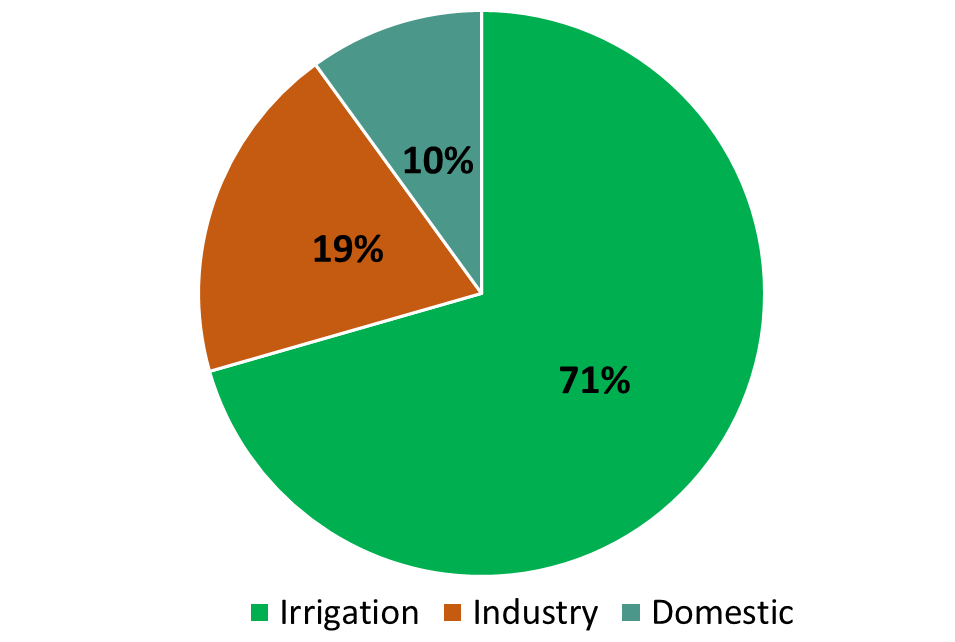


### Fig S5. Proportion (%) of irrigation, industrial and domestic water uses from 1970 to 2020.


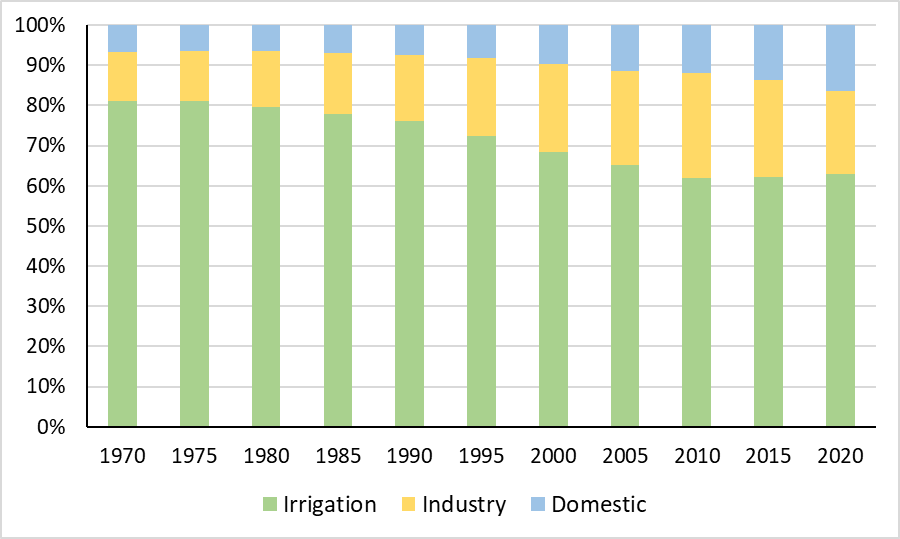


### Fig S6. Changes in water use structure (i.e., proportions of irrigation, industrial and domestic water use in TWU) in China from 1970 to 2020.


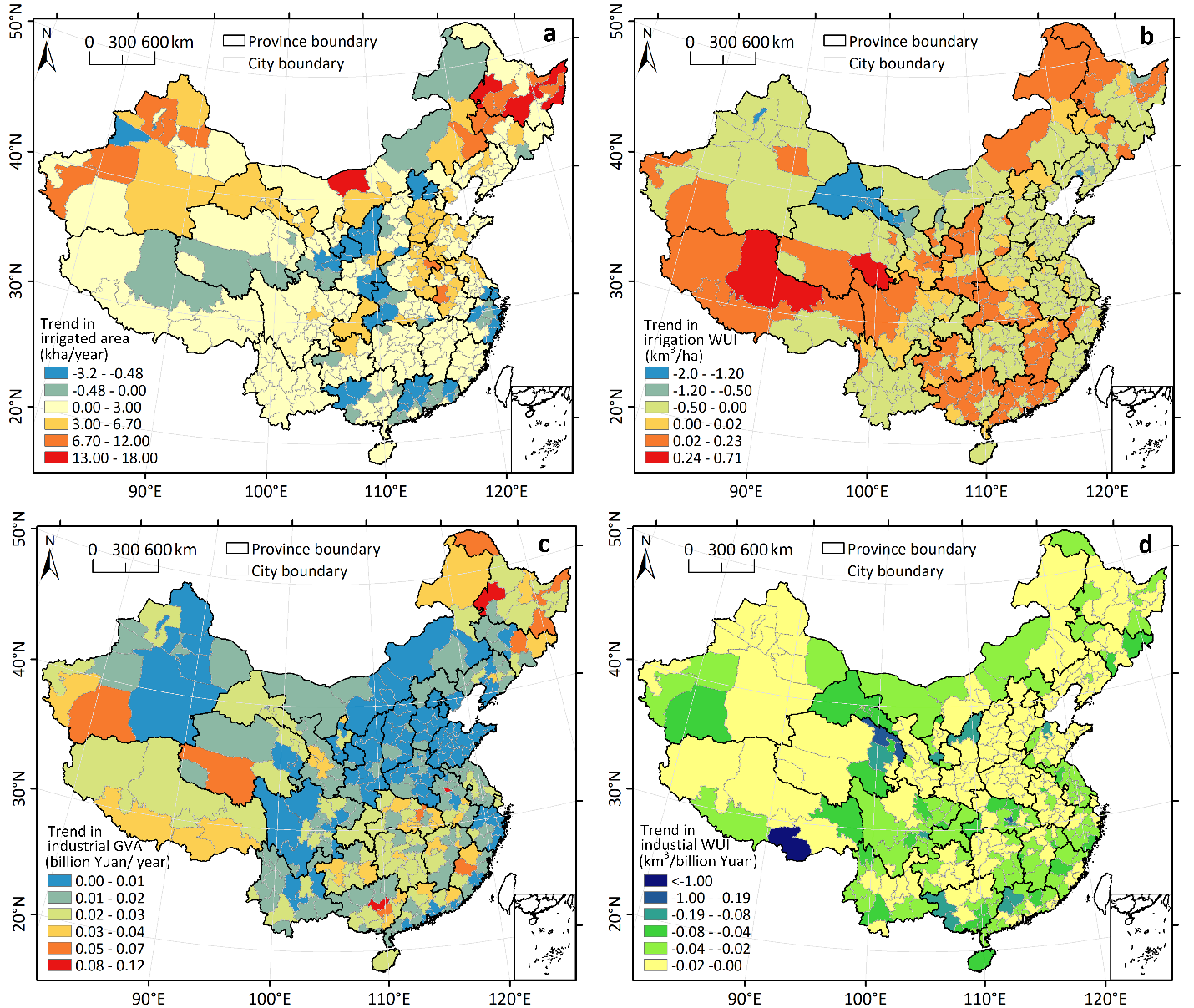


### Fig S7. Spatial patterns of trends in irrigated area (a), irrigation WUI (b), industrial GVA (c), industrial WUI (d), from 1970 to 2020. The map was generated using QGIS software, Version 3.40 (https://www.qgis.org).


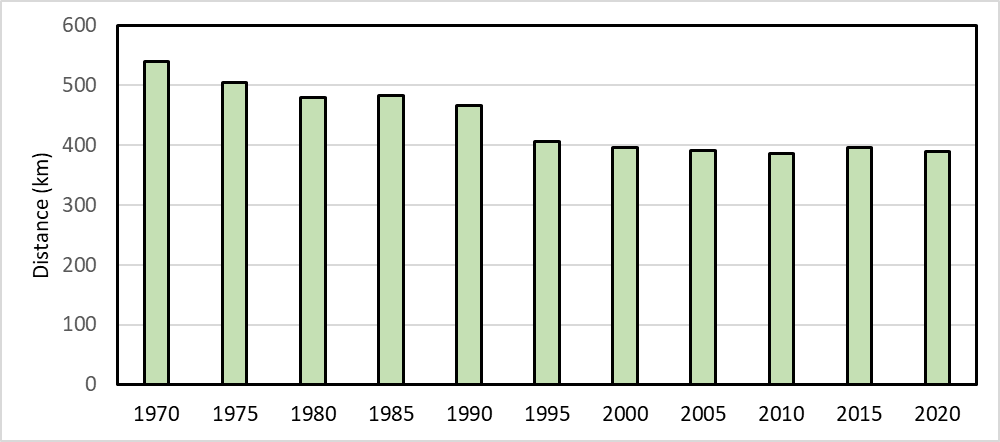


### Fig S8. Changes in spatial distance between gravity center of TWU and population from 1979 to 2020


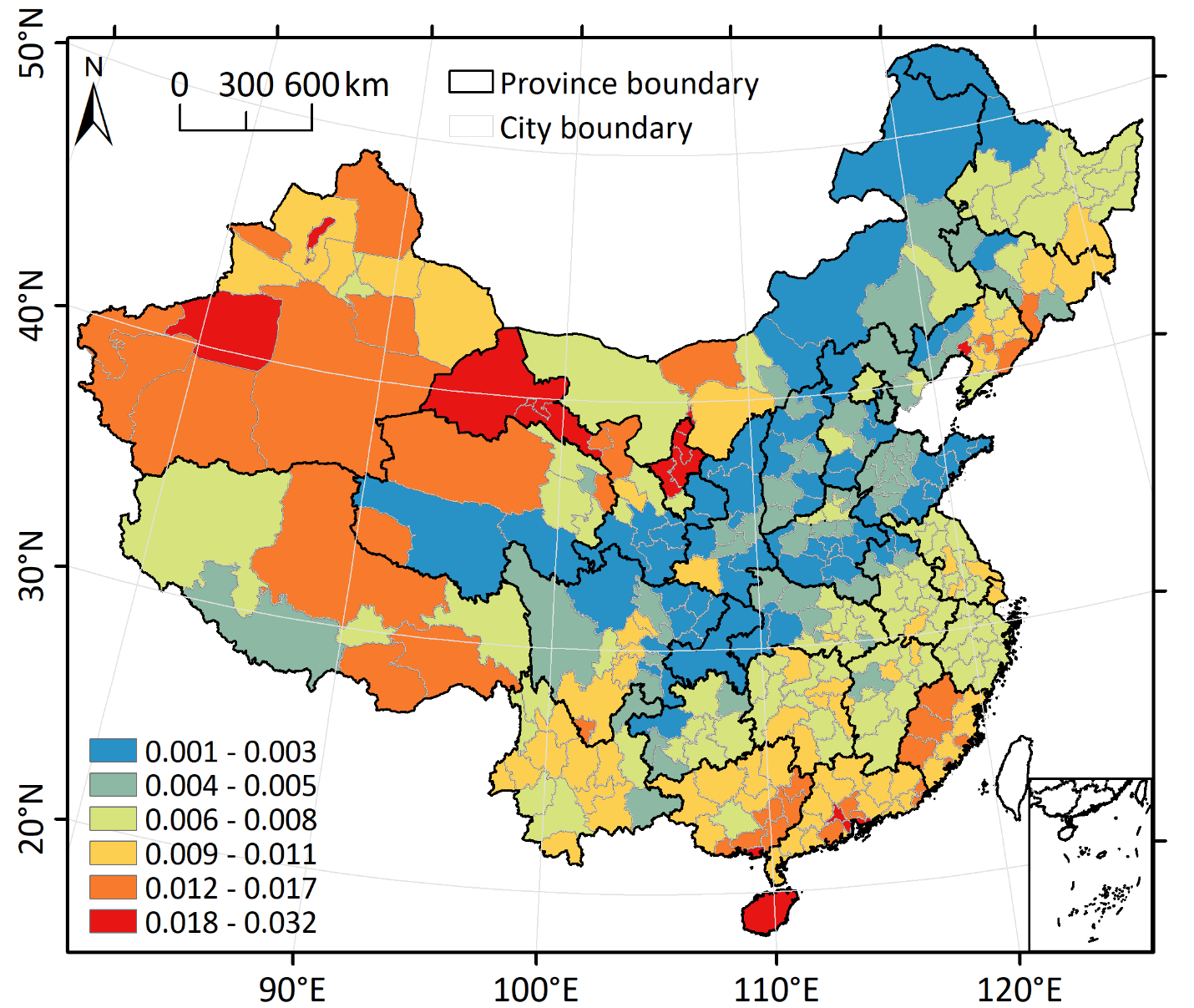


### Fig S9. Spatial distribution of average irrigation WUI in China over 1970 to 2020. The map was generated using QGIS software, Version 3.40 (https://www.qgis.org).

# 3. Supplementary Tabes

### Table S1. Sources of the water use and water availability data

| Id | Provinces | Temporal coverage of prefecture-level water use data | Sources |
| --- | --- | --- | --- |
| 1 | Beijing | 2000-2020 | <http://swj.beijing.gov.cn/> |
| 2 | Tianjin | 2000-2020 | <http://swj.tj.gov.cn/> |
| 3 | Hebei | 2000-2020 | <http://slt.hebei.gov.cn/>, Personal request |
| 4 | Shanxi | 2000-2020 | <http://slt.shanxi.gov.cn/>, [http://www.shanxi.gov.cn/sj/tjnj/](http://www.shanxi.gov.cn/sj/tjnj/%20%20) |
| 5 | Inner Mongolia | 2000-2020 | <http://slt.nmg.gov.cn/> |
| 6 | Liaoning | 2000-2020 | <http://slt.ln.gov.cn/> |
| 7 | Jilin | 2000-2020 | <http://slt.jl.gov.cn/>,  Personal request |
| 8 | Heilongjiang | 2000-2020 | [http://slt.hlj.gov.cn/](http://slt.hlj.gov.cn/%20) |
| 9 | Shanghai | 2000-2020 | [http://swj.sh.gov.cn/](http://swj.sh.gov.cn/%20) |
| 10 | Jiangsu | 2000-2020 | <http://jssslt.jiangsu.gov.cn>, Personal request |
| 11 | Zhejiang | 2000-2020 | [http://slt.zj.gov.cn/](http://slt.zj.gov.cn/%20) |
| 12 | Anhui | 2000-2020 | [http://slt.ah.gov.cn/](http://slt.ah.gov.cn/%20) |
| 13 | Fujian | 2000-2020 | [http://slt.fujian.gov.cn/](http://slt.fujian.gov.cn/%20) |
| 14 | Jiangxi | 2000-2020 | <http://slt.jiangxi.gov.cn/>, [http://tjj.jiangxi.gov.cn/](http://tjj.jiangxi.gov.cn/%20) |
| 15 | Shandong | 2000-2020 | <http://wr.shandong.gov.cn/>,  [https://tjj.shandong.gov.cn/](https://tjj.shandong.gov.cn/%20) |
| 16 | Henan | 2000-2020 | [https://slt.henan.gov.cn/](https://slt.henan.gov.cn/%20) |
| 17 | Hubei | 2000-2020 | [https://slt.hubei.gov.cn/](https://slt.hubei.gov.cn/%20) |
| 18 | Hunan | 2000-2020 | [http://slt.hunan.gov.cn/](http://slt.hunan.gov.cn/%20) |
| 19 | Guangdong | 2000-2020 | [http://slt.gd.gov.cn/](http://slt.gd.gov.cn/%20) |
| 20 | Guangxi | 2000-2020 | <http://slt.gxzf.gov.cn/>,  [http://tjj.gxzf.gov.cn/](http://tjj.gxzf.gov.cn/%20) |
| 21 | Hainan | 2000-2020 | <http://swt.hainan.gov.cn/>, Personal request |
| 22 | Chongqing | 2000-2020 | [http://slj.cq.gov.cn/](http://slj.cq.gov.cn/%20) |
| 23 | Sichuan | 2000-2020 | [http://slt.sc.gov.cn/](http://slt.sc.gov.cn/%20) |
| 24 | Guizhou | 2000-2020 | [http://mwr.guizhou.gov.cn/](http://mwr.guizhou.gov.cn/%20) |
| 25 | Yunnan | 2000-2020 | [http://wcb.yn.gov.cn/](http://wcb.yn.gov.cn/%20) |
| 26 | Tibet | 2000-2020 | [http://www.tpdc.ac.cn/zh-hans/](http://www.tpdc.ac.cn/zh-hans/%20) |
| 27 | Shaanxi | 2000-2020 | <http://slt.shaanxi.gov.cn/> |
| 28 | Gansu | 2000-2020 | <http://slt.gansu.gov.cn/> |
| 29 | Qinghai | 2000-2020 | <http://slt.qinghai.gov.cn/> |
| 30 | Ningxia | 2000-2020 | <http://slt.nx.gov.cn/> |
| 31 | Xinjiang | 2000-2020 | <http://slt.xinjiang.gov.cn/>,  <http://tjj.xinjiang.gov.cn/> |

Note: Beijing, Tianjin, Chongqing, Shanghai are the four municipalities directly under the Central Government of China.

### Table S2. Average annual sectoral water use of China for the period 2000-2020

| Sectoral water use | Mean annual value (10^8^ m^3^) | Proportion (%) |
| --- | --- | --- |
| Irrigation water use | 3317.8 | 57.2% |
| Industrial water use | 1239.3 | 21.4% |
| Domestic water use | 693.1 | 12.0% |
| Ecological water use | 119.0 | 2.1% |
| Other water use | 427.1 | 7.4% |

**References**

1. Shorrocks, A.F. Decomposition procedures for distributional analysis: a unified framework based on the Shapley value. *J. Econ. Inequal.* **11,** 99-126(2013).
2. Wan, G.H. Regression-based Inequality Decomposition: Pitfalls and a Solution Procedure. WIDER Discussion paper. *WIDER Working Paper Series* DP2002-101(2002).
3. Yao, S. On the decomposition of Gini coefficients by population class and income source: a spreadsheet approach and application. *Appl. Econ.* **10,** 1249-1264. DOI:10.1080/000368499323463(1999).
